# Supplementary material for: MetaRibo-Seq measures translation in microbiomes
Source: Nat Commun. 2020 Jun 29;11:3268. doi: 10.1038/s41467-020-17081-z (PMC7324362; doi:10.1038/s41467-020-17081-z)
Supplement: Supplementary file 10 — Supplementary Data 7 [file 41467_2020_17081_MOESM10_ESM.zip › File2/Confidence_High_Taxonomy/80942_out.krona.html]

Javascript must be enabled to view this page.

members
magnitude
magnitudeUnassigned
count
unassigned
taxon
rank

80942\_out

3

superkingdom
3
2

phylum
2
1239

1
186801
class

186802
1
order

family
1
186803

1
1213720
genus

species
796942
1

SRS893175\_contig\_number\_contig-100\_6623.93260

class
1
91061

1
186826
order

1
1300
family

1
1301
genus


SRS046496\_contig\_number\_contig-100\_1940.92532
1
1318
species

1
1224
phylum

class
1236
1

order
1
135625

1
712
family

1
724
genus


SRS893301\_contig\_number\_5184
species
712310
1
